# Supplementary material for: Validation and Clinical Application of the Japanese Version of the Patient-Reported Experience Measures for Intermediate Care Services: A Cross-Sectional Study
Source: Healthcare (Basel). 2024 Mar 29;12(7):743. doi: 10.3390/healthcare12070743 (PMC11012065; doi:10.3390/healthcare12070743)
Supplement: Supplementary file 1 [file healthcare-12-00743-s001.zip › healthcare-2898784-supplementary.pdf]

<u>Reported experience scale of those who used medical institutions and rehabilitation facilities</u>

※*Care refers to care, nursing, care, treatment, treatment, and consideration.

Question	Answer<br>*Please enclose only one of the most applicable options with ○.		
1.Was the timing of admission reasonable?	Yes	No	
2. Were the staff able to give me all the information I needed about my condition and illness?	Yes	Do not know	No
3. Were you able to clearly identify your own care goals?<br>Ex)<br>・be able to move freely in one's room<br>・be able to go shopping	Yes	Do not know	No
What is your goal？<br>【 】			
4. Were you able to think together about your own goals for home care after discharge?	Yes-always	Yes-sometimes	No
5. Was the room or shared area where you were hospitalized/admitted clean?	Very clean	Fairly clean	Not very clean

↓Please also look forward to the next page.

Japanese PREM developer: Goto Yuko, Miura Hisayuki

6. Have you experience any discomfort or fear from other users or visitors during your hospitalization?	Yes	No			
7. Have the staff adequately answer your questions?	Yes - always	Yes - sometimes	I had no need to ask	No	
8. Were you able to trust the staff?	Yes - always	Yes - sometimes	No		
9. Have you been involved to your satisfaction in making decisions about care and treatment?	Yes	No			
10. Were you involved in the decision about when to return to your home?	Yes - definitely	Yes - to some extent	I did not need to be involved	No	
11. Have the staff think about your family and home situation when planning care at your residence?	Yes - definitely	Yes - to some extent	It was no necessary	No	Do not know
12. Have the staff give your family and close friends enough information to care for you?	Yes - definitely	Yes - to some extent	I did not want or need them to	No	

↓Please also look forward to the next page.

13. Have you able to discuss with the staff the need to introduce aids and medical equipment for residential treatment?<br>Ex of introduce aids)<br>- Walkers, or wheelchairs, etc.<br>Ex of medical equipment)<br>- Infusions, or gastrostomy, etc.	Yes	No – it was not necessary to discuss it	No – but I would have liked them to
14. The need to receive other care and support after hospital exit/exit is well referred to as staff.<br>You were able to speak.<br>Ex)<br>- Physician visiting therapy<br>- Visit Rehabilitation and Visit Nursing<br>- Regional erosion services,<br>- Lending a car, etc.	Yes	No – it was not necessary to discuss it	No – but I would have liked them to
15. Comprehensively, you were asked to feel that care was unintentionally received from the staff during hospitalization/entry.	Yes – always	Yes – sometimes	No

Queries on questionnaires are filing as follows.
Research and development longevity national institute for medical research center at-home medical and area medical cooperation propulsion unit office station

Japanese PREM developer: Goto Yuko, Miura Hisayuki
